# PAM clustering algorithm based on mutual information matrix for ATR-FTIR spectral feature selection and disease diagnosis

**DOI:** 10.1186/s12874-025-02667-2

**Published:** 2025-10-01

**Authors:** Francesca Condino, Maria Caterina Crocco, Rita Guzzi

**Affiliations:** 1 Department of Economics, Statistics and Finance “Giovanni Anania”, University of Calabria, Rende (CS), 87036 Italy; 2 Department of Physics, Molecular Biophysics Laboratory, University of Calabria, Rende (CS), 87036 Italy; 3 STAR Research Infrastructure, University of Calabria, Rende (CS), 87036 Italy; 4 CNR-Nanotec, Rende (CS), 87036 Italy

**Keywords:** Feature selection, Shannon entropy, Dependence, Clustering, Dissimilarity measure

## Abstract

The ATR-FTIR spectral data represent a valuable source of information in a wide range of pathologies, including neurological disorders, and can be used for disease discrimination. To this end, the identification of the potential spectral biomarkers among all possible candidates is needed, but the amount of information characterizing the spectral dataset and the presence of redundancy among data could make the selection of the more informative features cumbersome. Here, a novel approach is proposed to perform feature selection based on redundant information among spectral data. In particular, we consider the Partition Around Medoids algorithm based on a dissimilarity matrix obtained from mutual information measure, in order to obtain groups of variables (wavenumbers) having similar patterns of pairwise dependence. Indeed, an advantage of this grouping algorithm with respect to other more widely used clustering methods, is to facilitate the interpretation of results, since the centre of each cluster, the so-called medoid, corresponds to an observed data point. As a consequence, the obtained medoid can be considered as representative of the whole wavenumbers belonging to the same cluster and retained in the subsequent statistical methods for disease prediction. An application on real data is finally reported to show the ability of the proposed approach in discriminating between patients affected by multiple sclerosis and healthy subjects.

## Introduction

In recent years, the use of Attenuated Total Reflectance Fourier-Transform Infrared (ATR-FTIR) spectroscopy applied on biofluids, tissues, cells, and other biological materials, has become crucial in the analysis of chemical and biological samples (see, for example, Baker et al., [1], Theakstone et al., [31] or Guler et al., [11]).

In particular, the ATR-FTIR spectroscopy has emerged as a promising tool for unveiling molecular signatures associated with neurological disorders, as multiple sclerosis (MS) [4, 6, 33]. Indeed, the diagnosis of MS poses significant challenges due to its heterogeneous clinical form and the absence of a specific diagnostic test. Complementary diagnostic tools that offer molecular insights into the pathological processes associated with MS are needed, allowing for earlier and more accurate diagnosis. In this respect, ATR-FTIR spectroscopy on biofluids, such as blood and blood components, can unveil specific molecular signatures associated with MS pathology. Alterations in biomolecular components, including lipid profiles and protein secondary structures, can be identified in the spectra of biological samples from MS patients when compared to healthy controls (HC). These changes could be indicative of underlying pathological processes, including inflammation, de-myelination, and neurodegeneration, offering valuable insights into the molecular basis of the disease.

These kinds of data are often characterized by high similarity, complexity and dimensionality that needs a deep analysis for discerning signal from noise and enhancing the interpretability of spectroscopic results. This requires some statistical strategy for selection and feature extraction that also considers the link among data. Indeed, in the spectral data analysis it is fundamental to take into account the relation between features, since they are potentially involved in complex interplay of molecular vibrations. Therefore, a key concept is the statistical dependence among ATR-FTIR spectral data, and a multivariate approach is often preferred, as it allows to handle dependence among variables. When the final aim is the disease discrimination, a possible solution to take into account the redundancy among data is to use some variable reduction method, such as principal component analysis or partial least squares, as pre-processing step, before employing models for classification. These techniques project the original feature space onto a set of optimized directions to generate a new set of variables. An excessive number of original variables can negatively impact the selection of projection directions, reducing the effectiveness of projection methods when applied to spectral data [20]. Moreover, these methods do not allow for direct interpretation of the original variables, as they are represented indirectly through projections onto new feature spaces. Another approach often employed to reduce the number of features to consider involves the use of some regularization technique, such as LASSO, RIDGE, Elasic Net and their variants, which constrain or shrink the coefficient estimates. In this context, a crucial issue regards the choice of penality function [9], which may raise concerns when applied to high-dimensional problems [32]. Moreover, interpreting results in high-dimensional settings requires caution [12].

In this paper, a new approach is considered to handle both dependence among features and variable reduction. In particular, the mutual information (MI) is considered as a general measure of pairwise dependence, and the selection of the most informative features is performed by employing a clustering algorithm, based on MI, to the set of variables. This allows to find groups of features highly dependent among them and separated as much as possible from the others and leads to the clustering of the corresponding MI matrix, bringing to light the underlying block data structure, when present. A similar procedure is used by Kojadinovic [16], who proposes a MI based approach in the context of agglomerative clustering algorithms.

In general, the concept of clustering of dependence matrices is not new in the biological and medical context, and it is often employed on the correlation matrices. It is particularly frequent in the analysis of gene expression data, when the aim is to find groups of genes characterized by similar pattern of correlation (see, for example, Pancaldi et al., [25], Fang et al., [10], Menard et al., [22]). In this context, Kong et al. [17] propose a new feature screening procedure together with the use of distance correlation, while Jensch et al. [14] present a robust and sparse Ensemble approach for outlier detection and feature selection.

In the context of data obtained by ATR-FTIR spectroscopy, by applying clustering algorithms to MI matrix, it becomes possible to identify groups of spectral features exhibiting similar behaviour and representing the underlying molecular structures. This leads to synthesizing spectral data through the cluster’s representative object. The main advantage in considering MI rather than correlation matrices is the possibility to take into account all kinds of dependence, not only the linear one. Moreover, unlike classical dimensionality reduction techniques, such as principal component analysis, this approach offers a better interpretability of the results, supporting researchers in identifying the most important molecular signatures within complex spectra.

This paper is organized as follows. In “The ATR-FTIR spectral data: an overview” section a brief overview of the particular kind of data is given to contextualize the problem. The issue of dependence among data is introduced in “Measuring dependence: the mutual information” section, and some aspects related to the estimation are discussed. “Synthesizing spectra: the MI based PAM algorithm” section is devoted to describe the non hierarchical clustering algorithm used to perform the clustering of the MI matrix, allowing for variable selection. Here, a dissimilarity measure among variables is proposed. Finally, an application to real data is shown and discussed in “The case study” section.

## The ATR-FTIR spectral data: an overview

ATR-FTIR has become a promising diagnostic tool in medicine, offering a rapid and non-invasive method for analyzing biological samples. Using the unique spectral signatures of biomolecules, this technique allows for the identification of specific molecular changes associated with different disease such as different form of cancers [29], COVID-19 [2, 34] and neurological disorders [26]. ATR-FTIR operates by directing infrared light into a crystal that has a high refractive index, which permits measurement of absorption bands from samples that are directly touching the crystal surface. The sample provides information about molecular vibrations and structural characteristics due to the interaction of the evanescent wave generated during this process. A series of peaks are present in the resulting spectrum, each representing specific functional groups and molecular conformations, which makes it possible to identify changes that may be related to pathological states at the molecular level [1]. In the context of disease diagnosis, ATR-FTIR spectral data can be employed to analyze a variety of biological materials, including tissues, blood, and other body fluids [1, 31]. Spectral features can be used to identify changes in biomolecular composition and conformation that indicate disease states when examined by researchers. In particular, shift in peak position or intensity variation of specific bands can reflect changes in structure or concentration associated with specific diseases, such as cancer [29] or neurodegenerative disorders [4, 6, 26]. Diagnostic applications can benefit from the multiple advantages of this experimental technique, including minimal sample preparation, real-time analysis, and the capability to perform on a wide range of sample types. Therefore, research on clinical application of ATR-FTIR spectroscopy can be considered as a complement to traditional diagnostic methods, enhancing sensitivity and specificity in early detection of diseases. In this study, infrared spectra of samples and solvent as background were recorded under the same experimental condition between 4000 and 900 cm${}^{-1}$. In this spectral range the vibrational absorption of specific functional groups of proteins and lipids contained in the plasma can be found. In particular, in the 1800-900 cm${}^{-1}$ region it can be found the overlapping absorption bands of proteins (amide I and amide II bands centred at about 1650 and 1545 cm${}^{-1}$) as well as some lower intensity peaks assigned to lipids [1]. The intensity of the absorption peaks is related to the concentration of the specific molecular components. To capture differences between the two groups of subjects, MS patients and HC individuals, the average spectra of the two groups can be compared. However, very often, the experimental signals are very similar and computational approaches with in-depth data analysis is often essential to highlight differences that may be associated with the presence of the disease. In this respect, the intensity and the wavenumbers of the absorption spectra can be considered as variables. The number of wavenumbers and then of the variables depends on the spectral resolution, 4 cm${}^{-1}$ in our data acquisition. We assume that each wavenumber can be described by a continuous random variable $X_j$, $j=1,...,p$, having probability density function $f (x_j)$ and support $\mathcal {X}_j$.

## Measuring dependence: the mutual information

The ATR-FTIR data are frequently characterized by the presence of a certain amount of redundancy, since they are often highly dependent on each other. The simplest and most widely used way to measure this redundancy is to compute the Pearson correlation coefficient. It is worth noting that this measure only quantifies the linear dependence among wavenumbers, and could be null also in the presence of strong dependence. It tends to oversimplify the complex relationships in ATR-FTIR spectra due to its linear and noise-sensitive assumptions. For spectral data analysis, where non-monotonic and in general complex biochemical interactions are common, more robust and flexible measures of dependence could be more suitable. With the aim to take into account both linear and non linear dependence, we consider a different measure of dependence, the so-called mutual information (MI). In contrast with the Pearson correlation, the MI is a measure able to detect all kinds of dependence and it is null only if the considered random variables are independent.

The meaning of the MI is strictly related to that of Shannon entropy [30], a central concept in information theory. Here, we just remind some basic notion for continuous random variables, highlighting that many of these concepts have a discrete counterpart, even if important differences between the two cases remain, such as the possibility of recording negative entropy values in continuous case (for details, see, e.g., Michalowicz, [23]).

### Definitions and notation

Formally, given a continuous random variable *X* having probability density function $f (\cdot )$ and support $\mathcal {X}$, the corresponding Shannon entropy, also called continuous or differential entropy, is given by:

$$\begin{aligned} H(X)= -\int _{\mathcal {X}} f(x) \log f(x) dx \end{aligned}$$

where *log* refers to the natural logarithm, so that we measure the entropy in *nats*. Otherwise, when the logarithm base is 2, the entropy is measured in bits. This quantity is connected with the amount of information held in a certain random variable and can be viewed as the expected value of the self-information, reflecting the amount in uncertainty about the variable *X*.

The eventual reduction in uncertainty due to the knowledge of a second random variable, connected with the first one, is exactly the MI. Indeed, given two continuous random variables $X_j$ and $X_{j'}$ with joint density $f(x_j,x_{j'})$, the MI between $X_j$ an $X_{j'}$ is given by:

1 $$\begin{aligned} I(X_j,X_{j'})=\int _{\mathcal {X}_{j'}} \int _{\mathcal {X}_j} f(x_j,x_{j'})\log \frac{f(x_j,x_{j'})}{f(x_j) f(x_{j'}) } dx_j dx_{j'}. \end{aligned}$$

This quantity can be viewed as the discrepancy, measured in terms of Kullback-Leibler divergence, between the joint distribution and the distribution under the hypothesis of independence between $X_j$ and $X_{j'}$, given by the marginals’ product. It becomes less the closer the two variables are to being independent. In particular, it is easy to verify that, in case of independence between $X_j$ and $X_{j'}$, the expression in (1) is equal to zero, since the ratio $\frac{f(x_j,x_{j'})}{f(x_j) f(x_{j'}) }$ is 1 everywhere.

Therefore, the case of a null MI corresponds to the case in which no reduction in uncertainty about one variable is provided by the knowledge of the other one. Conversely, this quantity increases as the relation between the two variables becomes stronger and the amount of information that a random variable contains about another increases. Ultimately, it can be shown that the MI is a symmetric measure and it is always non-negative, assuming a zero value if and only if $X_j$ and $X_{j'}$ are statistically independent, meaning that knowing one variable provides no information about the other. It quantifies the degree of dependence between two continuous random variables by measuring how much knowing one reduces the uncertainty about the other.

It is easy to verify that the MI can be also written in terms of differential entropy. Indeed, remembering that, given two random variables $X_j$ and $X_{j'}$, the marginals differential entropies and the joint differential entropy are given respectively by:

$$\begin{aligned} H(X_l)=-\int _ {\mathcal {X}_l} f(x_l) \log f(x_l) dx_l \ \ \ \text{for}\ \ \ l=1,2 \end{aligned}$$

and

$$\begin{aligned} H(X_j; X_{j'})=-\int _ {\mathcal {X}_{j'}} \int _ {\mathcal {X}_{j}} f(x_j, x_{j'}) \log f(x_j, x_{j'}) dx_j dx_{j'} \end{aligned}$$

from (1) the MI is given by:

$$\begin{aligned} I(X_j; X_{j'}) = H(X_j) + H(X_{j'}) - H(X_j, X_{j'}) \end{aligned}$$

All the previous concepts can be extended to the case of multiple random variables, but in the present work we ever will consider the case of pair of variables. For further details, see Cover and Thomas, [5].

### Estimating MI

The estimation of Shannon entropy and MI is quite simple in the case of discrete random variables, while it becomes quite complex in the context of continuous random variables.

When the family of distribution of the considered random variables is known, it is sometimes possible to obtain the expressions in closed form for both differential entropy and MI. This is the case, for example, of a bivariate normal distribution [5] having correlation $\rho$ and standard deviations $\sigma$, for which the differential entropies are:

$$\begin{aligned} H(X_l)=0.5 \cdot \log (2\pi e) \sigma ^2 \end{aligned}$$

for $l=j,j'$ and the MI is

$$\begin{aligned} I(X_j, X_{j'})=-0.5 \log (1-\rho ^2). \end{aligned}$$

In this case, even if the distribution is characterized by unknown parameters, they can be estimated and plugged into the previous expressions.

In most cases, the computation of these estimators in closed form is not so easy and some other method must be applied to obtain the estimates. Different proposal are present in literature, and one of the most widely used in practice involves the discretization of the random variable, aimed to partition the variable support into finite intervals and actually tracing back to discrete case [7]. Other approach involves B-splines [8] or kernel density estimations [24] to non-parametrically estimate the probability density function of the continuous variables and, consequently, to obtain the marginal entropies, the joint entropy and the MI. Another common procedure considers *k*-nearest neighbors statistics [18], in which the MI is obtained by considering local neighborhoods in the data space. It calculates distances to the $k-$nearest neighbors for both joint and marginal distributions. A more recent approach involves the copula theory, since it can be proved that the MI can be viewed as the entropy of the copula changed in sign [21]. It is worth noting that, regardless the method employed to obtain the MI estimates, a critical aspect regards the results’ admissibility, since not always the implemented procedure guarantees the non negativity of the fitted MI.

## Synthesizing spectra: the MI based PAM algorithm

In this paper, the Partition Around Medoids (PAM) method [15] is proposed, to find groups of variables having a similar pairwise pattern of dependence and to select the most relevant wavenumbers, reducing redundancy. This represents a different perspective with respect to the usual clustering task, which typically aims to group objects, as in this case the variables (wavenumbers) are clustered instead. One of the main advantages of the PAM algorithm is that the centre of each cluster, called medoid, corresponds to an observed member of dataset. Indeed, like the widely-used *k*-means algorithm, it is a non-hierarchical algorithm, able to furnish a partition of the data in a prefixed number of groups, but differently to the latter, the obtained centres correspond to observed members of the dataset or actual data points.

The algorithm is characterized by two distinctive phases: the so-called *build* and *swap* phases. The *build* phase initializes the clustering process by selecting *G* medoids from the dataset and assigning each data point to the nearest medoid, thereby forming the initial clusters and calculating the total partition cost based on the sum of dissimilarities between data points and their assigned medoids. The *swap* phase is directed to evaluate potential replacements for each medoid, by considering new candidates medoid and by verifying an eventual decrease in the total cost. If a candidate medoid results in a lower cost, it replaces the current medoid, leading to updated assignments and cost calculations. This process continues until no further improvement in cost is possible, indicating that an optimal or near-optimal clustering configuration has been achieved.

A key concept in performing the PAM algorithm is the definition of the cost function that needs the choice of a dissimilarity measure among units. Given the aims of this work, we choose to consider the MI among two random variables $I(X_j,X_{j'})$ ($j,j'=1,...,p$) as a similarity measure between each pair of wavenumbers, so that, the higher is the dependence among $X_j$ and $X_{j'}$, the stronger is considered the similarity between them. This leads to compose groups made up of highly dependent wavenumbers.

As highlighted by Kraskov and Grassberger [18], the definition of a dissimilarity measure based on MI become problematic when the involved variables are continuous. In order to define the dissimilarity to be used in the PAM algorithm, we consider a dissimilarity measure based on a normalized version of MI, already used by Kojadinovic [16] in the context of clustering and obtained through the following strictly increasing transformation:

2 $$\begin{aligned} I_{norm}(X_j,X_{j'})=\sqrt{(1-\exp (-2 \cdot I(X_j,X_{j'}))}. \end{aligned}$$

Consequently, the associated dissimilarity measure can be obtained as the complement to one of the suggested normalized similarity index, as follows:

3 $$\begin{aligned} d(X_j,X_{j'})=1-I_{norm}(X_j,X_{j'}). \end{aligned}$$

This proposed dissimilarity assumes values in the unit intervals, reaching its maximum value, equal to 1, in the case of independence between $X_j$ an $X_{j'}$. Therefore, by considering each couple of wavenumbers, we obtain a proper dissimilarity matrix, with null elements on the principal diagonal, and non negative values elsewhere.

Having defined the dissimilarity matrix, the PAM algorithm is performed to partition the set of the *p* wavenumbers points into *G* clusters such that the total dissimilarity is minimized. In this way, each cluster will contain wavenumbers having similar patterns of dependence and as related as possible. Here, the cost function is given by the sum of dissimilarities in (3) between each wavenumber and its nearest medoid. The *build* phase is then performed to obtain the initial set of medoids $m_1, m_2, ..., m_G$. This can be done randomly or based on certain criteria, like selecting the most centrally located points for each group. In the original version proposed by Kaufman and Rousseeuw [15] an initial partition is obtained by the successive selection of the *G* medoids. The first is the most centrally located in the set of objects, so that the sum of the dissimilarities to all other objects is as small as possible. Subsequently, the next medoids are individuated as those that decrease the objective function as much as possible. In this way, the first set of representative wavenumbers is identified and each other wavenumber is assigned to the cluster associated with the nearest medoid. In the following *swap* phase, the method allows to evaluate, for each cluster, potential new medoids by considering each non-medoid wavenumbers as a candidate. A swap of the current medoid with the candidate medoid is made if, by computing the cost associated with the possible switch between the current medoid and the candidate medoid, for all possible new medoids, there exists a different configuration that minimizes the total cost. If a better configuration is found, the current set of medoids is updated with the new representative wavenumbers and another possible swap is considered.

The algorithm terminates when the representative wavenumbers stabilize, meaning further swaps do not improve the total cost, or after a pre-defined number of iterations. A schematic representation of the algorithm is given in Algorithm 1, following the procedure described in Kaufman and Rousseeuw [15].

**Figure:**
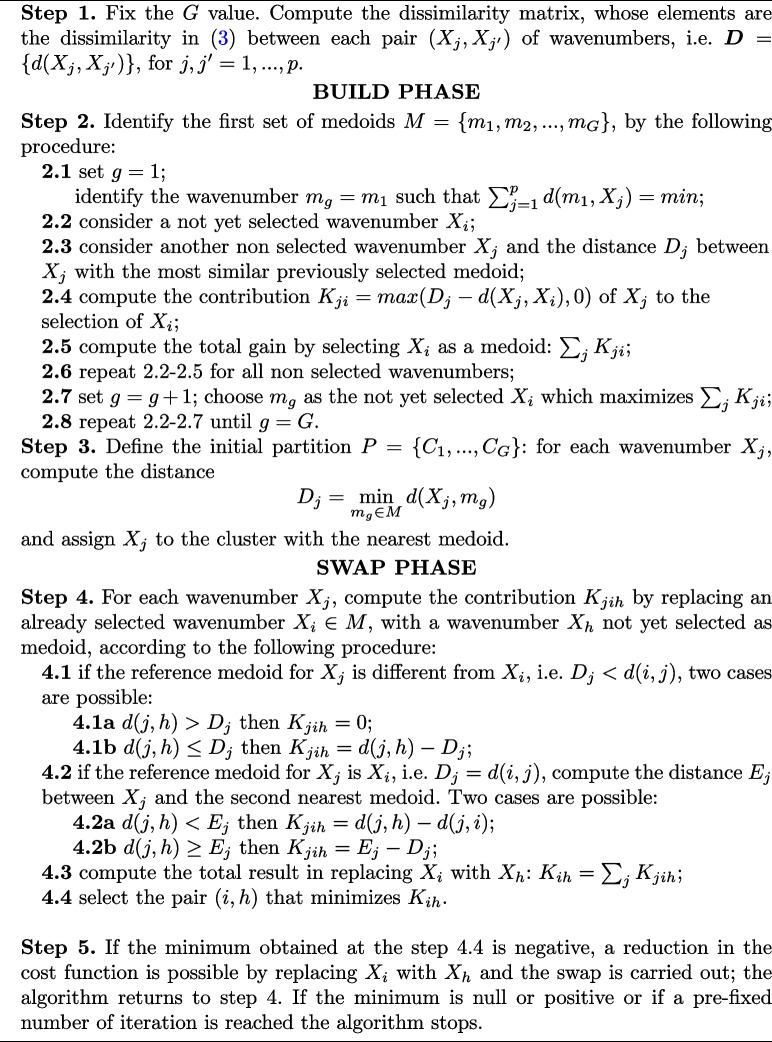
**Algorithm 1 **The clustering algorithm

At the end of the procedure, each cluster will be represented by an actual data point and this is particularly useful in our context, since the specific wavenumber selected as group medoid can be considered as representative for that cluster. In this way, we obtain a dimension reduction, going from *p* to *G* wavenumbers and only this limited number of variables will be considered in the subsequent applications of methods for disease prediction.

## The case study

To show the potentiality of the proposed methodology, in this work we consider data consisting of spectral features registered on the plasma of 85 subjects, including 45 MS patients and 40 healthy controls, recruited from April 2017 to July 2018 in the MS Center of the Annunziata Hospital in Cosenza, Italy. Details on the demographic information of this data were reported in Crocco et al. [6], where the FTIR absorption spectra were analyzed both in the fingerprint region ($1800-900 \text{cm}^{-1}$) and in the high region ($3050-2800 \text{cm}^{-1}$) and properly pre-processed before applying different univariate and multivariate statistical techniques aimed to discriminate patients from control subjects. Further details on pre-processing procedures can be found in Crocco et al. [6]. In the present analysis, we consider the spectral data of the fingerprint region, consisting of intensity values recorded for 631 different wavenumbers for the 85 subjects. All the subsequent statistical analysis was performed in R software.

### The normalized mutual information matrix clustering

In order to obtain the MI estimates in (1), here we consider a non parametric approach, based on $k-$nearest neighbour statistics. In particular, the estimator proposed by Kraskov et al. [18] and implemented in R package varrank is used. This method is particularly useful when MI must be estimated from finite samples in high-dimensional settings [18].

Having computed the MI matrix, whose elements correspond to pairwise MI between each couple of wavenumbers, it is possible to obtain the normalized measure of similarity by using the transformation in (2) and, consequently, the dissimilarity matrix with elements obtained using expression (3).

**Fig. 1:**
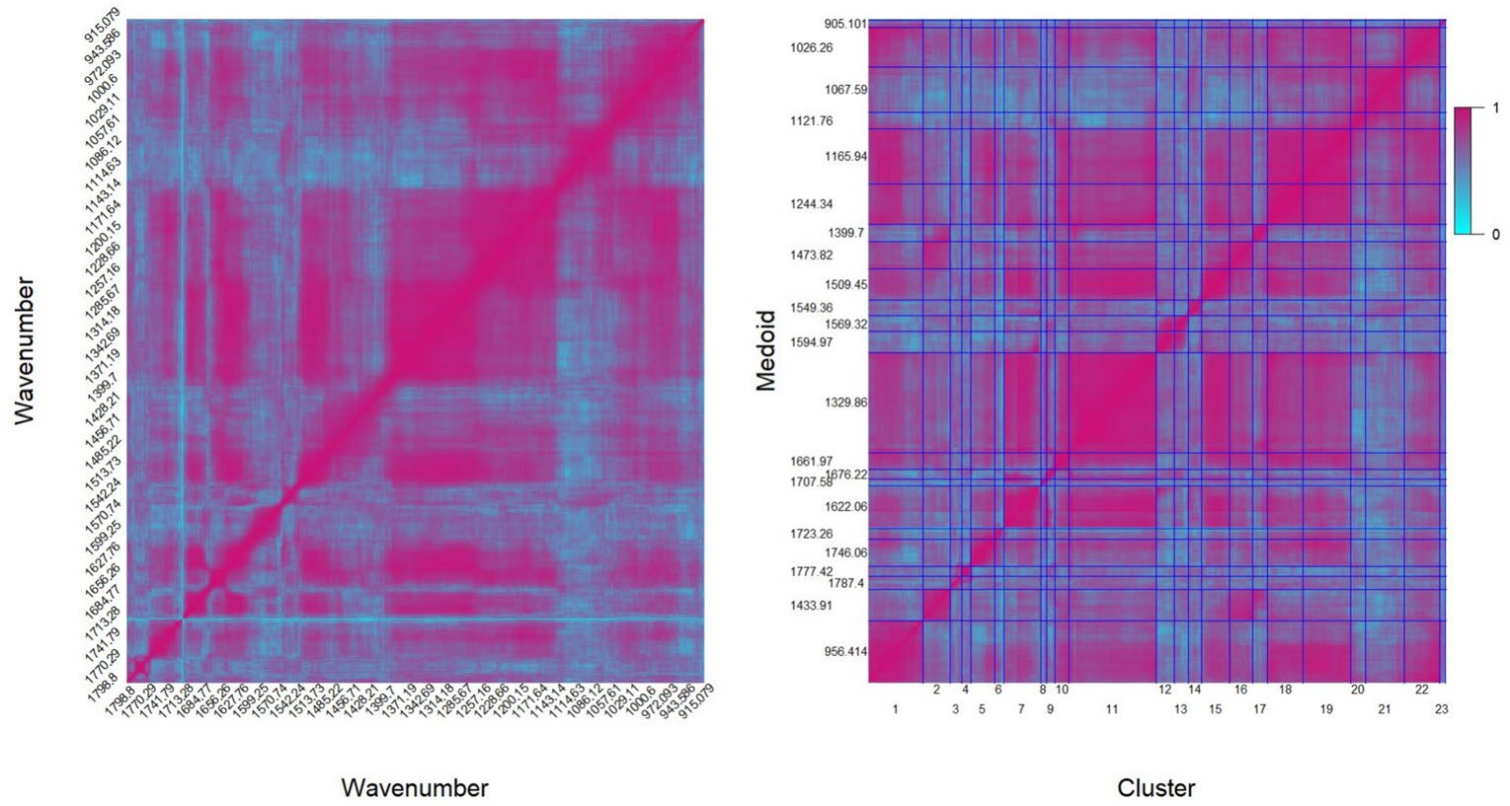
Heatmap of the normalized MI matrix for the fingerprint region, before (left panel) and after (right panel) the clustering

As it is expected, the observed pattern of the normalized MI for the available data is quite peculiar, as it can be seen by the heatmap reported in the left panel of Fig. 1. Specifically, the presence of possible groups of ATR-FTIR spectral data is evident, since different blocks of wavenumbers are characterized by high dependence, while others exhibit lower dependence. This evidence suggests that some exploratory method, such as clustering analysis, can help bringing to light this grouping data structure and identifying a representative element for each cluster able to synthesize block information.

**Fig. 2:**
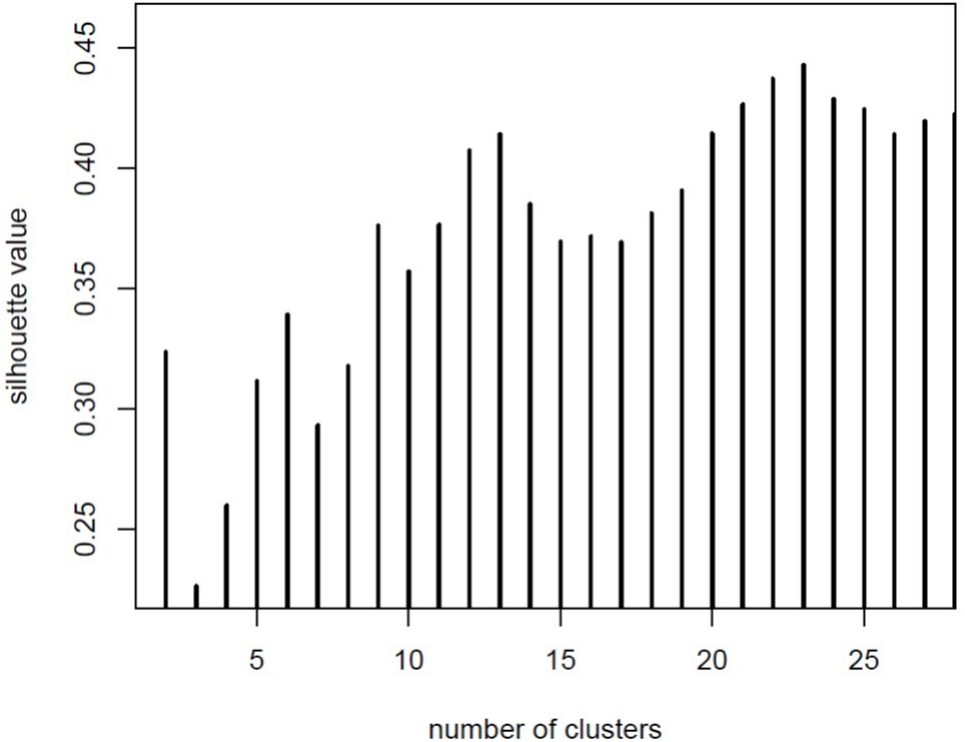
Silhouette values for different number of clusters

In order to have indications regarding the possible appropriate number of clusters to fix, we evaluate the behaviour of the silhouette statistic [28] in a reasonable range of values. The obtained results suggest to partition the wavenumbers in 23 clusters, as it can be assessed by the maximum value of the silhouette plot given in Fig. 2. Therefore, we apply the PAM algorithm described in the previous section, starting from the estimated dissimilarity matrix *D* and by fixing $G=23$.

The obtained partition of normalized MI matrix is graphically shown in the right panel of Fig. 1, where the specific representative set of wavenumbers, namely the medoids $m_1, m_2, ..., m_{23}$, is reported in ordinate, and related to the corresponding cluster, given in abscissa. From this plot, it is also evident the different size of the obtained clusters and the block structure of MI among clusters, that suggests the different pairwise dependence structure between wavenumbers belonging to different groups. In particular, all the squares around the diagonal are deep pink coloured, indicating a high level of dependence among wavenumbers belonging to the same cluster. This is also confirmed by the numerical results reported in Table 1, by the minimum and average level of $I_{norm}$ recorded in each cluster. The squares outside the principal diagonal are more or less pink coloured according to the level of dependence among wavenumbers belonging to different groups.

**Table 1:** Some characteristics for the 23 different obtained clusters

Cluster ID	Medoid	Cluster size	Min $I_{norm}$	Average $I_{norm}$
1	956.414	59	0.768	0.941
2	1433.91	30	0.777	0.926
3	1787.4	12	0.820	0.896
4	1777.42	10	0.936	0.972
5	1746.06	26	0.813	0.940
6	1723.26	10	0.760	0.925
7	1622.06	41	0.525	0.941
8	1707.58	6	0.662	0.910
9	1676.22	9	0.884	0.948
10	1661.97	16	0.906	0.970
11	1329.86	95	0.920	0.967
12	1594.97	20	0.825	0.954
13	1569.32	15	0.819	0.950
14	1549.36	15	0.865	0.936
15	1509.45	30	0.862	0.963
16	1473.82	26	0.911	0.948
17	1399.7	16	0.905	0.952
18	1244.34	39	0.949	0.974
19	1165.94	52	0.915	0.966
20	1121.76	16	0.922	0.965
21	1067.59	43	0.912	0.958
22	1026.26	38	0.913	0.956
23	905.101	7	0.818	0.907

**Fig. 3:**
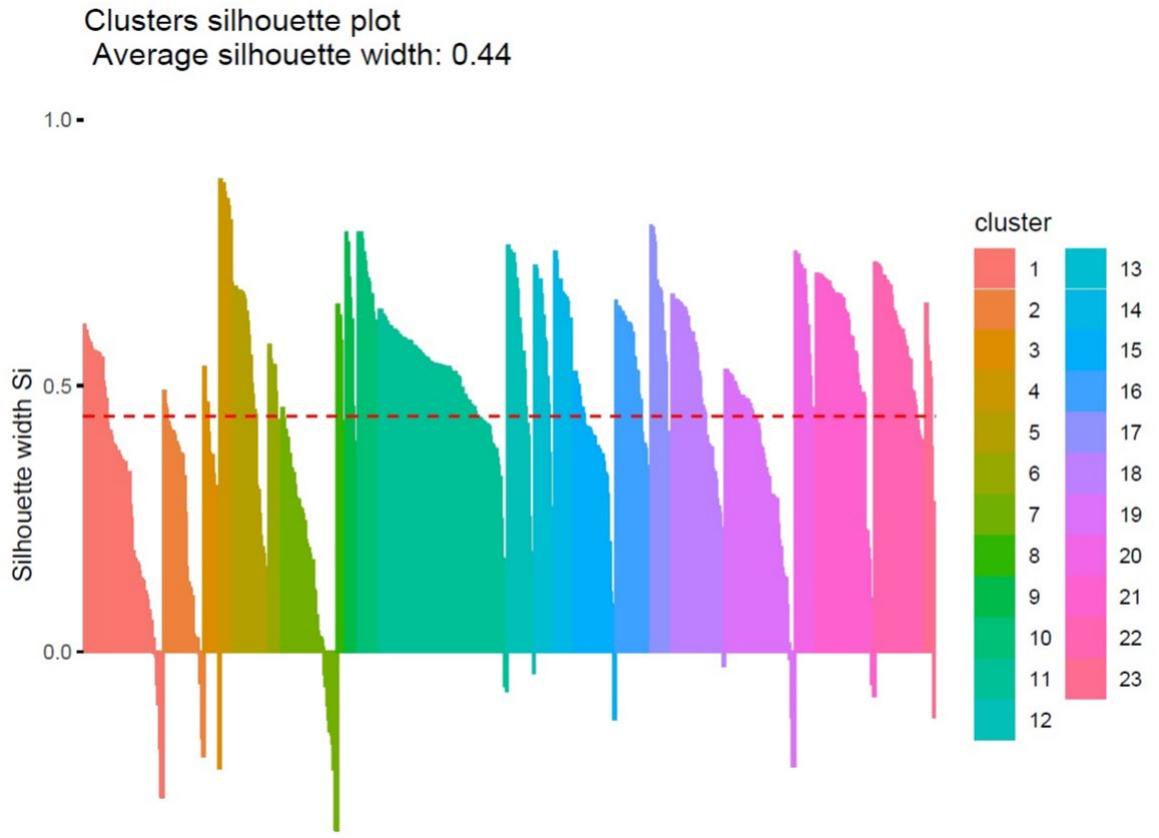
Silhouette values for different number of clusters

The silhouette values referred to the obtained partition and depicted in Fig. 3 shows in general a good cohesion of the clusters in relation to the separation level among neighbouring groups, with some exceptions revealed by the presence of negative values. Globally, the average silhouette width, equal to 0.44, reflects an appropriate clustering configuration and it can be reputed an acceptable value, also considering the complexity of the phenomenon under analysis.

### MS and controls discrimination

As mentioned in the previous sections, the medoids identified by the PAM algorithm can be considered representative of the whole set of wavenumbers belonging to the same cluster. Therefore, in the following, we will narrow the attention to them as possible covariates for performing the classification task aimed to distinguish between MS patients and controls.

**Table 2:** Median, minimum, and maximum wavenumber values in patients and control group, along with results from the Mann–Whitney U-test. The adjusted $p-$value, according to the Benjamini–Hochberg procedure, is reported in the last column

Wavenumber	Median in HC	Median in MS	Min	Max	*W statistic*	$p-$value	Adjusted p-value
956.414	0.0017	0.0021	0.0000	0.0027	558	0.0026	0.0152
1433.91	0.0311	0.0316	0.0255	0.0328	567	0.0034	0.0157
1787.4	0.0002	0.0002	0.0001	0.0002	856.5	0.7050	0.9008
1777.42	0.0003	0.0003	0.0002	0.0004	912	0.9194	0.9611
1746.06	0.0017	0.0019	0.0011	0.0036	691	0.0664	0.1273
1723.26	0.0005	0.0005	0.0002	0.0009	770	0.2542	0.3898
1622.06	0.0647	0.0626	0.0617	0.0670	1330	0.0002	0.0018
1707.58	0.0002	0.0002	0.0001	0.0004	782.5	0.3029	0.4355
1676.22	0.0389	0.0389	0.0376	0.0402	924	0.8361	0.9535
1661.97	0.0756	0.0744	0.0711	0.0779	1176	0.0153	0.0439
1329.86	0.0303	0.0318	0.0292	0.0337	581	0.0050	0.0193
1594.97	0.0429	0.0431	0.0403	0.0468	906	0.9614	0.9614
1569.32	0.0745	0.0750	0.0722	0.0776	701	0.0805	0.1425
1549.36	0.1243	0.1242	0.1214	0.1270	881	0.8706	0.9535
1509.45	0.0524	0.0535	0.0516	0.0548	634	0.0194	0.0446
1473.82	0.0265	0.0269	0.0244	0.0285	593	0.0070	0.0229
1399.7	0.0497	0.0509	0.0479	0.0530	300	$<0.0001$	$<0.0001$
1244.34	0.0291	0.0297	0.0282	0.0312	631	0.0181	0.0446
1165.94	0.0167	0.0172	0.0153	0.0184	674	0.0471	0.0985
1121.76	0.0162	0.0165	0.0140	0.0177	753	0.1971	0.3238
1067.59	0.0102	0.0101	0.0054	0.0130	979	0.4895	0.6622
1026.26	0.0105	0.0113	0.0090	0.0145	545	0.0018	0.0138
905.101	0.0001	0.0001	0.0000	0.0003	875.5	0.8326	0.9535

To have some initial indication regarding a possible role of the selected wavenumbers in discriminating between MS and control subjects, we perform a Mann-Whitney U-test to compare the median values between the two groups. The obtained results are given in Table 2, along with the $p-$values adjusted for multiple testing according to the Benjamini-Hochberg procedure [3], with a target FDR of 0.05, as implemented in R. The obtained results confirm that there exists a significant difference between the medians for many of the selected wavenumbers, suggesting a possible importance in the discrimination process, even after controlling for the risk of false positives due to multiple testing. Nevertheless, it is important to consider the joint effect of the covariates.

**Fig. 4:**
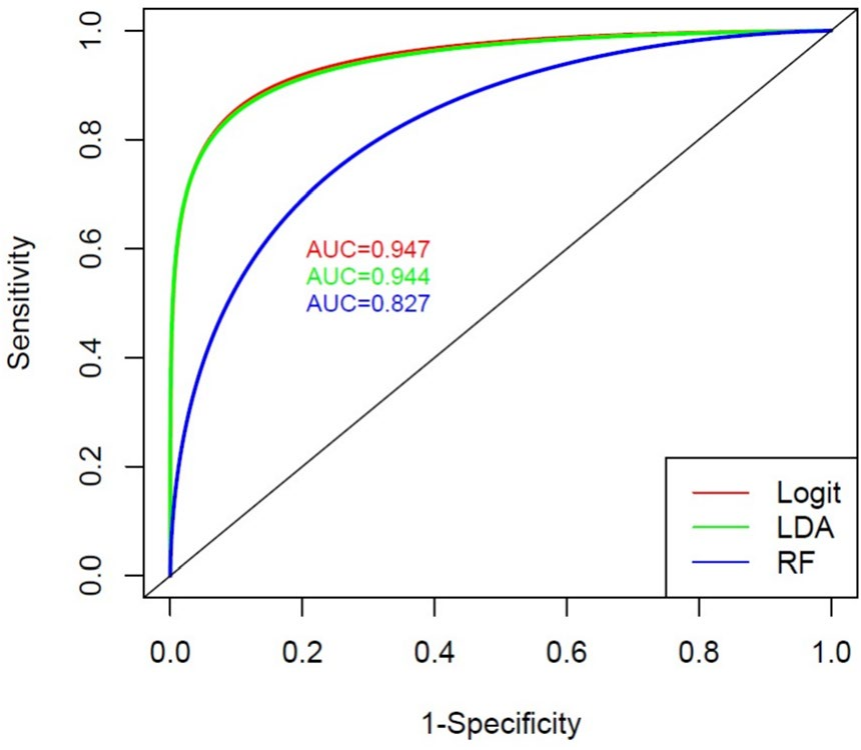
Receiver Operating Curve (ROC) for logit regression and linear discriminant analysis for fingerprint region

Therefore, these wavenumbers are employed as covariates in some of the most widely used multivariate models for classification. In particular, logistic regression, linear discriminant analysis (LDA) and random forests (RF) are considered to discriminate between MS and controls. As suggested by the usual measures of diagnostic performance reported in Table 3, all the three chosen methods show good performance in separating the two groups of individuals. The lower accuracy is recorded when the RF method is applied, due to a poorer ability in detecting the true negative cases. Moreover, the Receiver Operating Characteristic (ROC) curves in Fig. 4 show higher values of the area under the curves (AUC) for logit and LDA models, confirming a better ability of these methods in distinguishing between MS and HC.

**Table 3:** Subjects classification and measures of diagnostic performance according to logit model, linear discriminant analysis (LDA) and random forest (RF) methods

Observed	Predicted		Measures of diagnostic accuracy	
	MS	HC		
**Logit**				
MS	40	5	Sensitivity	88.89%
HC	5	35	Specificity	87.50%
Tot	45	40	Accuracy	88.24%
**LDA**				
MS	39	6	Sensitivity	86.67%
HC	3	37	Specificity	92.50%
Tot	42	43	Accuracy	89.41%
**RF**				
MS	38	7	Sensitivity	84.44%
HC	13	27	Specificity	67.50%
Tot	51	34	Accuracy	76.47%

**Fig. 5:**
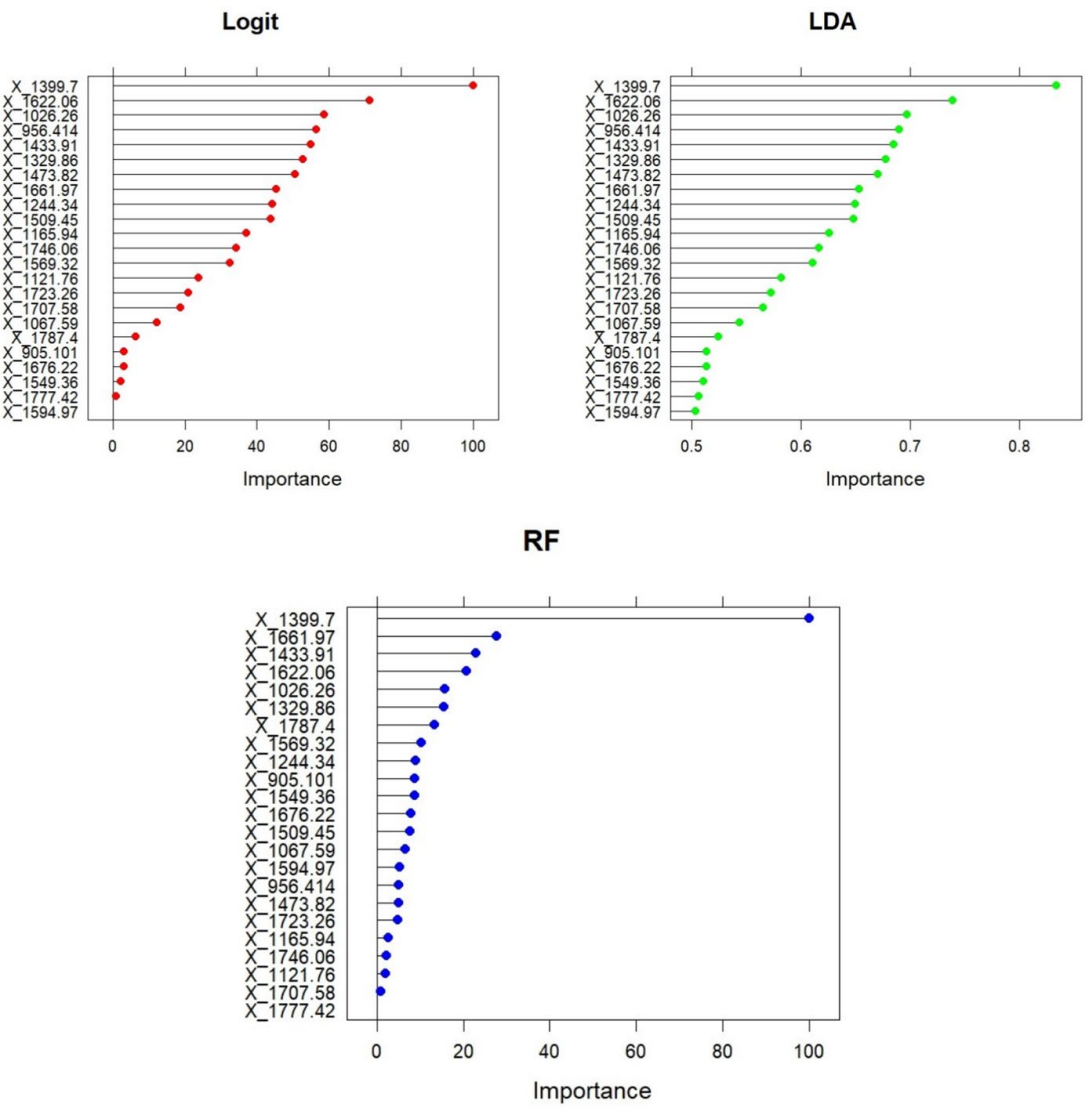
Boxplot of the area under the curve (AUC), sensitivity (Sens) and specificity (Spec) for the repeated *k*-fold cross-validation data. Box stretches from first quartile $Q_1$ to third quartile $Q_3$ and the median is shown as a line across each box. The values lower than $Q_1-1.5*IQR$ or upper than $Q_1+1.5*IQR$, where *IQR* is the interquartile range, are shown as black dots. The whiskers show the lower and upper values, excluding outliers

Given the limited number of available subjects, we consider all data as training set avoiding to reserve some of them as test set, so that it is not possible to directly estimate the test error rate. Anyway, we consider the *k*-fold cross-validation technique to estimate this quantity using the available training data [13]. The procedure was implemented using the caret package [19], and the models were trained using the default tuning parameter settings, where applicable. The obtained results in terms of AUC, sensitivity and specificity on 10-time repeated 5-fold cross validation data for the three models are summarized in Fig. 5. Moreover, to evaluate the potentiality of the proposed approach with respect to some classical techniques, these results were compared with those obtained using methods already employed on the second derivatives of the same spectra [6], as well as with an elastic-net regularized logit model. Furthermore, to highlight the advantage of using MI as a dependence measure, a comparison is made with the results obtained by partitioning the Pearson’s correlation matrix through the PAM algorithm. The descriptive statistics of the resulting AUC values for the repeated 5-fold cross-validation data, shown in Table 4, suggest that discrimination methods based on MI and medoids are competitive and often perform better on average than the other methods. As expected, given the presence of some non-monotonic relationships among wavenumbers, the procedure based on MI outperforms the use of Pearson’s correlation, being in these cases more suitable to capture the strength of association.

**Table 4:** Descriptive statistics of the area under the curve (AUC), for the repeated *k*-fold cross-validation data, using different methods

Method	Min	$Q_1$	Median	Mean	SD	$Q_3$	Max
Logit (MI & medoids)	0.583	0.722	0.799	0.795	0.102	0.875	1.000
LDA (MI & medoids)	0.667	0.767	0.847	0.841	0.083	0.899	1.000
RF (MI & medoids)	0.569	0.771	0.861	0.842	0.084	0.899	0.972
PCA-LDA	0.514	0.653	0.722	0.733	0.117	0.819	0.972
RF	0.583	0.764	0.840	0.833	0.105	0.917	1.000
PLS-DA	0.528	0.681	0.764	0.762	0.097	0.830	0.944
Logit-ENET	0.500	0.750	0.826	0.820	0.100	0.889	1.000
Logit (Pearson’s corr. & medoids)	0.611	0.722	0.778	0.789	0.091	0.861	0.986
LDA (Pearson’s corr. & medoids)	0.569	0.781	0.840	0.831	0.099	0.899	0.986
RF (Pearson’s corr. & medoids)	0.556	0.750	0.837	0.814	0.109	0.889	0.986

**Fig. 6:**
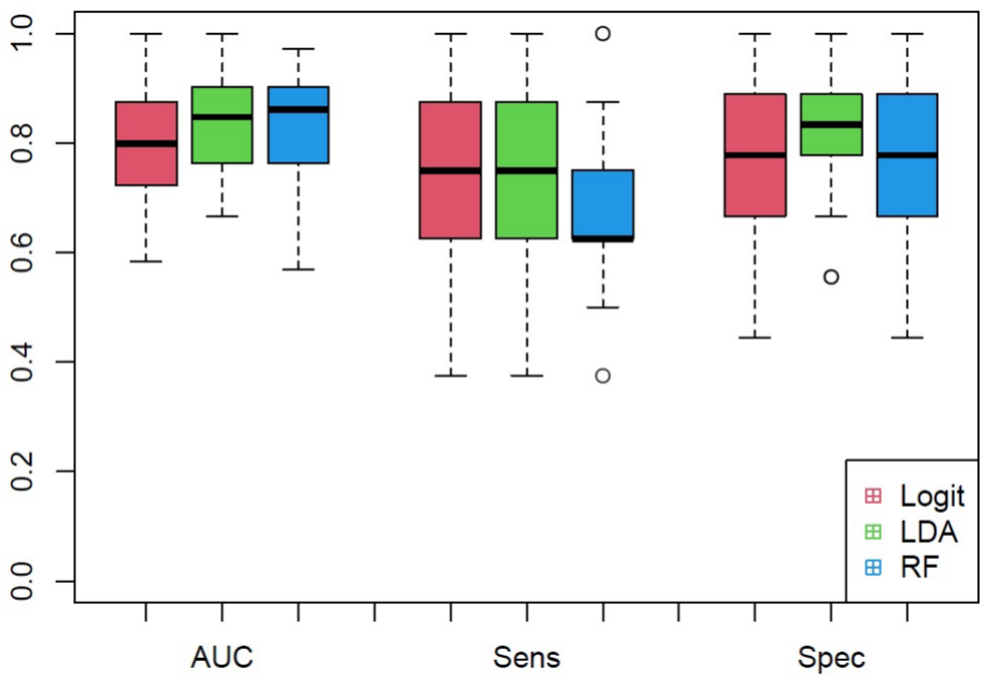
Variable importance of medoids for the three models

To obtain a measure of the variable importance and to evaluate the impact of each wavenumber on the discrimination process, it is possible to use the function *varImp* in the caret package. Depending on the considered method, this function characterize the general effect of predictors on the model in a different way (see Kuhn [19] for further details). The obtained variable importances referred to each of the fitted model for the 23 different medoids are shown in Fig. 6. It is worth to note that the wavenumber “1399.7” is the most important variable for all the three considered methods. This wavenumber is assigned to symmetric stretching vibration of COO- group of fatty acids (i.e. lipid molecules) and amino acids (i.e. constituent units of proteins) as well as to symmetric bending modes of methyl groups of proteins [27]. So that the selected variable is close related to the molecular components (lipids and proteins) that are mostly affected in MS disease [6].

## Conclusions

The diagnosis and monitoring of diseases, in particular those highly disabling and difficult to identify, such as many of the neurodegenerative disorders, has been the main focus of different researches. Clinical diagnostic purposes can benefit greatly from the powerful biomolecular profiling method ATR-FTIR spectroscopy of easily collected body fluids, as long as the experimental data are properly analysed and interpreted.

In the present work, a new approach aimed to efficiently synthesize the available information from ATR-FTIR data is proposed. In particular, the method deals with the variable selection issue as a pre-processing stage in disease discrimination problems. To this end, the PAM algorithm is performed on the basis of a dissimilarity measure defined starting from the concept of MI. Indeed, the particular kind of data requires to take into account the dependence among variables, both in terms of linear and non linear relationship. Therefore, the MI is taken as a measure of similarity between variables and properly transformed to obtain an associated measure of dissimilarity. The so performed PAM algorithm leads to identify a specific block structure characterizing the MI matrix and, contextually, to select a set of representative wavenumbers for the blocks, corresponding to the set of the obtained medoids. It is worth to note that, the selected variables often corresponds to specific functional groups of molecular components such as lipids and proteins that are affected in MS. In this sense, this kind of approach allows to perform feature selection, with the final aim to consider a limited number of covariates to use in a second step, where models for subject classification are employed. The results obtained on real data clearly show the potentiality of this approach. All the considered models for classification task reach high levels of accuracy and performance, as suggested by the usual diagnostic tools. It is important to highlight that these estimates may overstate the ability of these models, as they are fitted and tested on the same data. Therefore, although the limited number of available subjects does not allow for a direct estimate of the test error rate, we consider the cross-validation technique. The obtained results suggest that the proposed method is likely to perform well on independent data, also in comparison with other approaches. It is worth noting that the logit model performs very well on the training data, but shows a slight drop in performance on not used for training, as indicated by the cross-validation results. One potential limitation of the proposed strategy lies in its scalability, as its computational cost may increase significantly with the size of the dataset. In this case an extension of PAM algorithm, the so called CLARA (Clustering Large Applications) [15], could be employed to deal the large number of wavenumbers, in order to reduce computing time.

Finally, it is important to highlight that the proposed method is potentially suitable to be employed in very different contexts, wherever highly redundant variables are present and a possible block structure in the mutual information matrix can be hypothesized. In this view, the possibility to explore the performance of the proposed approach in different application fields represents a natural perspective for the future.

## Data Availability

The raw data generated and analyzed during the current study are available from the corresponding author on reasonable request.

## References

[1] Baker MJ, Trevisan J, Bassan P, Bhargava R, Butler HJ, Dorling KM, Fielden PR, Fogarty SW, Fullwood NJ, Heys KA, Hughes C, Lasch P, Martin-Hirsch PL, Obinaju B, Sockalingum GD, Sulé-Suso J, Strong RJ, Walsh MJ, Wood BR, Gardner P, Martin FL. Using Fourier transform IR spectroscopy to analyze biological materials. Nat Protoc. 2014;9(8):1771–91. 24992094 10.1038/nprot.2014.110 PMC4480339

[2] Banerjee A, Gokhale A, Bankar R, Palanivel V, Salkar A, Robinson H, Shastri JS, Agrawal S, Hartel G, Hill MM, Srivastava S. Rapid classification of COVID-19 severity by ATR-FTIR spectroscopy of plasma samples. Anal Chem. 2021;93(30):10391–6. 34279898 10.1021/acs.analchem.1c00596

[3] Benjamini Y, Hochberg Y. Controlling the false discovery rate: a practical and powerful approach to multiple testing. J R Stat Soc Ser B Stat Methodol. 1995;57:289–300.

[4] Condino F, Crocco MC, Pirritano D, Petrone A, Del Giudice F, Guzzi R. A linear predictor based on FTIR spectral biomarkers improves disease diagnosis classification: an application to multiple sclerosis. J Pers Med. 2023;13: 1596. 10.3390/jpm13111596. 38003911 10.3390/jpm13111596 PMC10672539

[5] Cover TM, Thomas JA. Element of information theory. 2nd ed. John Wiley & Sons; 2006.

[6] Crocco MC, Moyano MFH, Annesi F, Bruno R, Pirritano D, Del Giudice F, Petrone A, Condino F, Guzzi R. ATR-FTIR spectroscopy of plasma supported by multivariate analysis discriminates multiple sclerosis disease. Sci Rep. 2023;13: 2565. 36782055 10.1038/s41598-023-29617-6 PMC9924868

[7] Darbellay GA, Vajda I. Estimation of the information by an adaptive partitioning of the observation space. IEEE Trans Inf Theory. 1999;45:1315–21.

[8] Daub CO, Steuer R, Selbig J, et al. Daub CO, Steuer R, Selbig J, Kloska S. Estimating mutual information using B-spline functions - an improved similarity measure for analysing gene expression data. BMC Bioinformatics. 2004;31(5):118. 10.1186/1471-2105-5-118. 10.1186/1471-2105-5-118 PMC516800 15339346

[9] Fan J, Li R. Variable selection via nonconcave penalized likelihood and its oracle properties. J Am Stat Assoc. 2001;96:1348–60.

[10] Fang J, Ma Q, Chu C, Huang B, Li L, Cai P, Batista PJ, Tolentino KEM, Xu J, Li R, Du P, Qu K, Chang HY. Pirch-seq: functional classification of non-coding RNAs associated with distinct histone modifications. Genome Biol. 2019;20: 292. 31862000 10.1186/s13059-019-1880-3 PMC6924075

[11] Güler G, Guven U, Oktem G. Characterization of human prostate cancer stem cells with ATR-FTIR spectroscopy. Analyst. 2019;144:2138–49. 10.1039/c9an00093c 30742170

[12] Hastie T, Tibshirani R, Friedman J. The Elements of Statistical Learning: Data Mining, Inference, and Prediction. 2nd ed. Berlin: Springer; 2017.

[13] James G, Witten D, Hastie T, Tibshirani R. An Introduction to Statistical Learning: with Applications in R. Springer; 2013.

[14] Jensch A, Lopes MB, Vinga S, Radde N. ROSIE: RObust sparse ensemble for outlIEr detection and gene selection in cancer omics data. Stat Methods Med Res. 2022;31(5):947–58. 35072570 10.1177/09622802211072456 PMC9014683

[15] Kaufman L, Rousseeuw PJ. Partitioning Around Medoids (Program PAM). In: Kaufman L, Rousseeuw PJ, editors. Finding Groups in Data. John Wiley & Sons, Inc., Hoboken, New Jersey; 1990.

[16] Kojadinovic I. Agglomerative hierarchical clustering of continuous variables based on mutual information. Comput Stat Data Anal. 2004;46:269–94.

[17] Kong J, Wang S, Wahba G. Using distance covariance for improved variable selection with application to learning genetic risk models. Stat Med. 2015;34:1708–20. 25640961 10.1002/sim.6441 PMC4441212

[18] Kraskov A, Stogbauer H, Grassberger P. Estimating mutual information. Phys Rev E. 2004;69:1–16. 10.1103/PhysRevE.69.066138 15244698

[19] Kuhn M. Building predictive models in R using the caret package. J Stat Softw. 2008;28(5):1–26.27774042

[20] Liu W, Li Q. An efficient elastic net with regression coefficients method for variable selection of spectrum data. PLoS One. 201712(2): e0171122. 10.1371/journal.pone.0171122 28152003 10.1371/journal.pone.0171122 PMC5289531

[21] Ma J, Sun Z. Mutual information is copula entropy. Tsinghua Sci Technol. 2011;16(1):51–4.

[22] Ménard C, Dulong J, Benjamin DR, Hebraud, Verdière L, Pangault C, Sibut V, Bezier I, Bescher N, Monvoisin C, Gadelorge M, Bertheuil N, Flécher E, Casteilla L, Collas P, Sensebé L, Bourin P, Eszpagnolle N, Tarte K. Integrated Transcriptomic, Phenotypic, and Functional Study Reveals Tissue-Specific Immune Properties of Mesenchymal Stromal Cells. Stem Cells. 2020;38(1):146-59. 10.1002/stem.3077 31502731

[23] Michalowicz JV, Bucholtz F, Nichols JM. Handbook of differential entropy. 1st ed. New York: Chapman and Hall/CRC; 2013.

[24] Moon Y, Rajagopalan B, Lall U. Estimation of mutual information using kernel density estimators. Phys Rev E. 1995;52(3):2318–21. 10.1103/physreve.52.2318 9963673

[25] Pancaldi V, Schubert F, Bähler J. Meta-analysis of genome regulation and expression variability across hundreds of environmental and genetic perturbations in fission yeast. Mol Biosyst. 2010;6(3):543–52. 20174682 10.1039/b913876p

[26] Paraskevaidi M, Morais CLM, Lima KMG, Snowden JS, Saxon JA, Richardson AMT, Jones M, Mann DMA, Allsop D, Martin-Hirsch PL, Martin FL. Differential diagnosis of Alzheimer’s disease using spectrochemical analysis of blood. Proc Natl Acad Sci U S A. 2017;114(38):E7929-38. 28874525 10.1073/pnas.1701517114 PMC5617251

[27] Rehman IU, Movasaghi Z, Rehman S. FTIR and Raman characteristic peak frequencies in biological studies. In: Vibrational Spectroscopy for Tissue Analysis. CRC Press; 2012.

[28] Rousseeuw PJ. Silhouettes: a graphical aid to the interpretation and validation of cluster analysis. J Comput Appl Math. 1987;20:53–65.

[29] Sala A, Anderson DJ, Brennan PM, Butler HJ, Cameron JM, Jenkinson MD, Rinaldi C, Theakstone AG, Baker MJ. Biofluid diagnostics by FTIR spectroscopy: A platform technology for cancer detection. Cancer Lett. 2020;477:122–30. 32112901 10.1016/j.canlet.2020.02.020

[30] Shannon CE. A mathematical theory of communication. Bell Syst Tech J. 1948;27(4):623–56.

[31] Theakstone AG, Rinaldi C, Butler HJ, Cameron JM, Confield LR, Rutherford SH, Sala A, Sangamnerkar S, Baker MJ. Fourier-transform infrared spectroscopy of biofluids: a practical approach. Transl Biophotonics. 2021;3: e202000025.

[32] Wang X, Park T, Carriere KC. Variable selection via combined penalization for high-dimensional data analysis. Comput Stat Data Anal. 2010;54(10):2230–43.

[33] Yonar D, Ocek L, Tiftikcioglu BI, Zorlu Y, Severcan F. Relapsing-remitting multiple sclerosis diagnosis from cerebrospinal fluids via Fourier transform infrared spectroscopy coupled with multivariate analysis. Sci Rep. 2018;8: 1025. 29348591 10.1038/s41598-018-19303-3 PMC5773569

[34] Zhang L, Xiao M, Wang Y, Peng S, Chen Y, Zhang D, Zhang D, Guo Y, Wang X, Luo H, Zhou Q, Xu Y. Fast screening and primary diagnosis of COVID-19 by ATR-FT-IR. Anal Chem. 2021;93(4):2191–9. 33427452 10.1021/acs.analchem.0c04049

